# The lncRNA *HMS* recruits RNA-binding protein HuR to stabilize the 3′-UTR of *HOXC10* mRNA

**DOI:** 10.1016/j.jbc.2021.100997

**Published:** 2021-07-22

**Authors:** Priyanka Priyanka, Madhur Sharma, Sanjeev Das, Sandeep Saxena

**Affiliations:** 1 DNA Replication and Cell Cycle Laboratory, National Institute of Immunology, New Delhi, India; 2 Departmeny of Biochemistry, UDSC, New Delhi, India; 3 Department of Biotechnology, JNU, New Delhi, India

**Keywords:** long noncoding RNA (long ncRNA, lncRNA), posttranscriptional regulation, RNA–protein interaction, cancer biology, cell biology, ELAV-like protein 1 (HuR (human antigen R)), ARE, AU-rich element, CNBP, CCHC-type zinc finger nucleic acid binding protein, DMEM, Dulbecco’s modified eagle’s medium, ELAV, embryonic lethal abnormal vision, FBS, fetal bovine serum, *HMS*, *HOXC10* mRNA stabilizing factor, HuR, human antigen R, lncRNA, long noncoding RNA, LUAD, lung adenocarcinoma, OS, osteosarcoma, RBP, RNA-binding protein, shRNA, short hairpin RNA, TCGA, The Cancer Genome Atlas, TSS, transcriptional start site

## Abstract

Long noncoding RNAs (lncRNAs) have been reported to drive key cancer pathways but the functions of majority of lncRNAs are unknown making a case for comprehensive functional evaluation of lncRNAs. With an aim to identify lncRNAs dysregulated in human cancers, we analyzed the cancer patient database of lung adenocarcinoma (LUAD), which revealed an upregulated lncRNA, *LINC02381* (renamed *HOXC10*mRNA stabilizing factor or *HMS* in this study), whose depletion results in proliferation defects and inhibition of colony formation of human cancer cells. In order to identify the binding targets of *HMS*, we screened for *cis*-genes and discovered that *HOXC10*, an oncogene, is downregulated in the absence of *HMS*. Depletion of *HMS* does not affect the *HOXC10* promoter activity but inhibits the *HOXC10* 3′-UTR-linked luciferase reporter activity. Since lncRNAs have been known to associate with RNA-binding proteins (RBPs) to stabilize mRNA transcripts, we screened for different RBPs and discovered that HuR, an ELAV family protein, stabilizes *HOXC10* mRNA. Using RNA pull-down and deletion mapping experiments, we show that HuR physically interacts with the cytosine-rich stretch of *HMS* and *HOXC10* 3′-UTR to stabilize *HOXC10* mRNA. *HOXC10* is overexpressed in many human cancers, and our discovery highlights that lncRNA *HMS* sustains the *HOXC10* mRNA levels to maintain the invasive phenotypes of cancer cells.

Long noncoding RNAs (lncRNAs) play a pivotal role in diverse physiological processes by gene regulation at the transcriptional and posttranscriptional levels (1, 2). For transcriptional regulation, lncRNAs recruit chromatin modifiers to promote epigenetic activation or silencing of gene expression. For example, lncRNA *ANRIL* recruits the polycomb repressive complex that mediates the transcriptional silencing by methylating the lysine 27 of histone H3 of the neighboring *CDKN2A* and *CDKN2B* genes (3). LncRNAs have also been reported to alter the DNA methylation of target genes: LncRNA *Dum* recruits Dnmt1, Dnmt3a, and Dnmt3b to the promoter of *DPPA2* gene, thereby silencing its expression and stimulating myogenic differentiation (4).

For posttranscriptional gene regulation, lncRNAs modulate mRNA splicing, mRNA turnover as well as translation and subcellular localization of target mRNAs (5, 6, 7, 8, 9). It is believed that partial base pairing with the target mRNA leads to mRNA decay while extended base pairing stabilizes the mRNA (10). Independent of base pairing with the target mRNA, lncRNAs may also function as decoys or scaffolds to alter binding of transcriptional regulatory factors (11). Posttranscriptional gene regulation by lncRNAs is mediated by associating with RNA-binding proteins (RBPs), which are known to modulate the expression, stability, maturation, and transport of target mRNAs (10). Different families of RBPs, primarily classified on the basis of their RNA-binding domains, are believed to associate with specific sequence motifs in the mRNA substrate, such as poly U, Poly C, poly G/U, and CA repeats (12). Stabilization of cyclin D1 mRNA by lncRNA *LAST* in cooperation with CCHC-type zinc finger nucleic acid binding protein (CNBP) highlights the role of lncRNA-RBP complex in the regulation of mRNA stability (13). While *LAST*-CNBP complex associated with the 5′UTR of cyclin D1 mRNA, the stabilization of *CDK6* mRNA by lncRNA *MYU* and RBP hnRNP-K complex is mediated at its 3′UTR (14). The versatility of such association is apparent from the role of lncRNA *Linc-RoR*, which either stabilizes or destabilizes the *c-Myc* mRNA, depending on whether it associates with RNP I or AUF1, respectively (15).

Among the well-characterized RBPs is the ubiquitously expressed embryonic lethal abnormal vision (ELAV) family protein HuR (the human antigen R/HuA) known to bind to the AU-rich elements (AREs) present at the 3′ UTR of its target mRNAs (16, 17). While association of RBPs such as AUF1 and ZFP36 leads to rapid RNA decay of ARE-containing transcripts, ELAV family proteins have an opposite effect: ELAV proteins prevent the association of destabilizing RBPs with mRNA transcript, thereby inhibiting their degradation (18, 19, 20). Though AREs are well accepted as the canonical sites for HuR association, in-depth studies find that HuR can also associate with many nonconserved sequences: Association of HuR has been observed with the polypyrimidine tract region located between the splice site and the branch point of pre-mRNA (21, 22).

Recent reports have highlighted the significance of LncRNAs-HuR association in diverse conditions ranging from normal development to human diseases (23, 24). The mechanism of lncRNAs regulating HuR activity is equally diverse: LncRNA *DUXAP10* physically interacts with HuR and suppresses the cytoplasm-nuclear translocation of HuR, while a macrophage-specific lncRNA MAARS tethers HuR in the nucleus preventing its cytosolic shuttling (8, 23). Another distinct example is lncRNA *ASB16-AS1*, which modulates the stability of HuR protein by recruiting the ubiquitin E3 ligase beta-TrCP1 (25). The role of lncRNAs in modulating the mRNA-stabilizing activity of HuR has received much attention: LncRNA *OIP5-AS1* physically associates with HuR as well as *MEF2C* mRNA transcript with which it shares partial complementarity resulting in the stabilization of *MEF2C* mRNA (24). Silencing of *OIP5-AS1* disrupts HuR binding to *MEF2C* mRNA implying that *OIP5-AS1* serves as a scaffold for HuR binding to *MEF2C* mRNA. This is in contrast to OIP5-AS1 preventing HuR binding to *CCND1* mRNA, exemplifying the disparate control of HuR activity by lncRNAs (26).

In this study, we report that a novel lncRNA, LINC02381 (referred as *HOXC10* mRNA stabilizing factor or *HMS* in this study) regulates the mRNA levels of homeobox C10 (*HOXC10*) oncogene.

*HOXC10* is a member of the homeobox gene family that also plays a vital role in embryonic development (27, 28, 29). Human cells encode a total of 39 HOX genes that are found in four clusters (HOXA, HOXB, HOXC, and HOXD) located on different chromosomes. The regulation of homeobox genes by lncRNAs is best exemplified by lncRNA *HOTAIR* that represses the transcription of HOXD locus by recruiting the polycomb chromatin complex 2, while another well-studied lncRNA, *HOTTIP* forms a complex with WDR5 and the histone methyltransferase protein MLL resulting in H3K4 methylation and transcriptional activation of the HOXA locus (30, 31). Many other lncRNAs are located in and associated with HOX gene clusters, but their role in HOX gene regulation is yet to be completely understood (32). LncRNA *HMS* was discovered in a screen devised in the present study to identify that lncRNAs dysregulated in human cancers are expressed 135 kb downstream from the HOXC locus. We attempted to divulge the mechanism by which *HMS* regulates *HOXC10* and discovered that it does not alter the transcription from *HOXC10* promoter but stabilizes the 3′UTR of *HOXC10* mRNA. *HMS* utilizes its polypyrimidine stretch to associate with the RNA-stabilizing protein, HuR and recruit it to the *HOXC10* mRNA. Thus, we report a novel lncRNA, *HMS*, functions as a *HOXC10* mRNA stabilizing factor by associating with the HuR to stabilize *HOXC10* mRNA, which has an essential role in the proliferation of cancer cells.

## Results

### An integrative analysis of the transcriptional profiles of The Cancer Genome Atlas (TCGA) cancer samples identifies dysregulated lncRNAs

**Figure 1:**
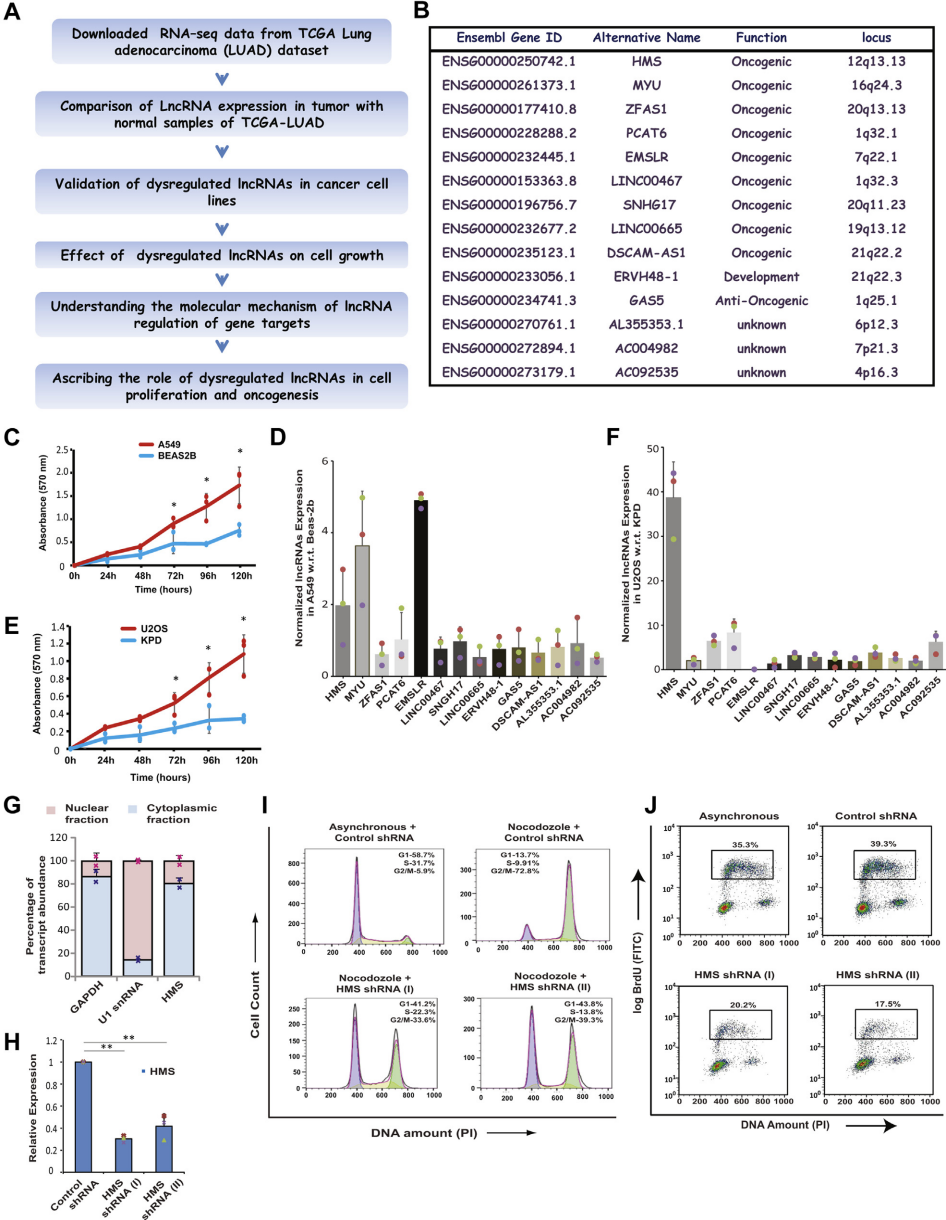
**Comparison of transcriptional profiles of TCGA cancer samples identifies dysregulated lncRNAs.***A*, schematic outline of the strategy employed for identification of dysregulated lncRNAs, their validation, identification of their gene targets and determining their effect on cell proliferation and oncogeneis. *B*, list of 14 selected lncRNAs. From the 224 upregulated lncRNAs, we selected 14 lncRNAs based on literature search, genomic screens of lncRNA function, proximity to *cis*-genes, and expression levels in various tissues. The table lists the Ensembl gene ID, alternate names, previously reported function and genomic locus. It should be noted that the screen identifies many lncRNAs across the genome that have been previously shown to have oncogenic activity. *C*, MTT proliferation assay to compare the growth rate of A549, an adenocarcinomic human alveolar epithelial cell line, with BEAS-2B, a nontumorigenic lung epithelial cell line, derived from a human lung tissue. The absorbance at 570 nm (minus the plate background absorbance at 630 nm) reflects the proportion of viable cells for each cell line at the indicated time interval. The data represents mean of three independent experiments ±standard deviation (S.D). Individual data points of the line chart have been shown as markers of the same colour. *p*-values calculated using two-tailed Student’s *t* test display that cell proliferation rate in A549 is significantly different from BEAS-2B samples at the indicated time intervals (∗*p* < 0.05). *D*, relative expression of 14 selected lncRNAs in A549 cells with respect to BEAS-2B cells, evaluated by individual quantitative real-time PCR. Beta-actin gene was used as the endogenous control for normalization of lncRNA expression in the two cell lines. The data represents mean of three independent experiments ±S.D. *E*, MTT proliferation assay to compare the growth rate of U2OS with KPD cell line as described in part *C* (n = 3, ∗*p* < 0.05, Student’s *t* test). *F*, evaluation of the levels of 14 lncRNAs in two human osteosarcoma cell: U2OS (rapid-proliferating, highly invasive) and KPD (slow-proliferating, less invasive) that have been previously used as contrasting pair of cell lines to study the role of ncRNAs in cancer. The data represents mean of three independent experiments ±S.D. *G*, subcellular fractionation of U2OS cells followed by evaluation of abundance of the indicated transcripts by qRT-PCR in nuclear and cytoplasmic fractions. The *bar graph* displays the percentage of the total amount of detected transcripts in different fractions. *GAPDH* and U1 snRNA serve as controls for cytoplasmic and nuclear fractions, respectively. The data is represented as mean ± SD from two independent experiments. Markers in *pink* and *blue color* point to individual data points of nuclear and cytoplasmic fractions, respectively. *H*, U2OS cells were transduced with lentiviral particles expressing either control shRNA or shRNAs targeting different regions of *HMS*: shRNA (I) and shRNA (II), followed by puromycin selection to obtain stable knockdown cells. The level of *HMS* was quantified by individual quantitative real-time PCR. Glyceraldehyde-3-phosphate dehydrogenase (*GAPDH*) was used as the endogenous control for normalization of *HMS* expression in different samples. The data represents mean of three independent experiments ±S.D. Note that both shRNA-I (∗∗*p* < 0.01, ANOVA/Tukey’s test) and shRNA-II (∗∗*p* < 0.01, ANOVA/Tukey’s test) significantly reduced *HMS* levels in comparison to control shRNA samples. *I*, depletion of *HMS* leads to an accumulation of cells in the G1 phase. U2OS cells stably depleted for *HMS*, as described in part *H*, were cultured in media with or without nocodazole for 16 h followed by cell cycle distribution analysis by flow cytometry. The inset shows the distribution of cells in different phases of the cell cycle. *J*, depletion of *HMS* impedes S phase progression. Flow cytometry of control or *HMS*-depleted U2OS cells, pulsed with BrdU. *Dot plot* displays BrdU incorporation (y-axis) and DNA content (x-axis). The *inset* shows the percentage of cells incorporating BrdU. Individual data points have been shown for all charts.

Comparative analysis of lncRNA alterations across 13 cancer types indicated that while majority of differentially expressed lncRNAs are highly cancer type specific, many lncRNAs have been discovered whose dysregulation is conserved across a few, if not all, cancer types (33). As described in Figure 1*A*, we followed a systematic approach for identification of dysregulated lncRNAs in lung adenocarcinoma (LUAD). We downloaded gene expression data of LUAD tumor and normal samples from the TANRIC portal, which annotates around 12,000 lncRNAs from the TCGA database. We selected the samples whose mRNA and miRNA expressions were available *via* UCSC Genome Browser. A differential expression analysis was performed on 420 tumor and 20 normal samples from LUAD to identify upregulated (fold_change > 2) or downregulated (fold_change < 0.5) lncRNAs. By doing so, 224 and 140 lncRNAs were observed to be upregulated and downregulated, respectively in LUAD samples compared with the normal samples. We investigated if these lncRNAs were dysregulated in other cancer types, searched for information in genome-wide screens of lncRNAs, analyzed their genomic locus, proximity to growth modulating genes, expression in various tissues, and confidence levels of the primary lncRNA transcript and consequently selected 14 lncRNAs (Fig. 1*B*). Many of these selected 14 lncRNAs are reported in the literature to have a role in cell proliferation and tumorigenicity. For example, lncRNA *MYU* functions downstream of Wnt/c-Myc signaling to promote tumorigenicity of colon cancer cells, lncRNA *ZFAS1* promotes metastasis of clear cell renal cell carcinoma by targeting the miR-10a/SKA1 pathway, lncRNA *EMSLR* induces tumorigenesis *via* the c-Myc pathway, and lncRNA *PCAT6* induces cell growth and metastasis *via* Wnt/β-catenin pathway (14, 34, 35, 36). Similarly, lncRNAs *DSCAM-AS1*, *LINC00467*, and *SNHG17* have been reported to promote breast, lung, and gastric cancer, respectively, highlighting the efficacy of our screen to identify dysregulated lncRNAs (37, 38, 39).

Next, we evaluated if the dysregulation of lncRNAs is preserved in established cell lines so that we could experimentally address the molecular mechanism of lncRNA function. Thus, we compared the levels of the 14 identified lncRNAs in A549, an aggressive lung adenocarcinoma cell line, with BEAS-2B, a nontumorigenic lung epithelial cell line. As expected the proliferation rate of A549 is significantly higher than that of BEAS-2B, and we observed that out of 14 lncRNAs tested, three lncRNAs, *HMS*, *MYU*, and *EMSLR*, were upregulated in A549 cells (Fig. 1, *C* and *D*). We have previously identified the role of miRNAs in osteosarcomas (OS), and we are now pursuing the role of lncRNAs but since OS patient data is not available at TCGA, we initiated our investigation in this study by identifying lncRNAs that are dysregulated across other cancer types and then assaying their dysregulation in OS (40, 41). Thus, we assayed if the upregulation of lncRNAs is conserved in osteosarcomas: Out of the three lncRNAs that were upregulated in A549 cell line, we observed that only *HMS* was significantly upregulated in aggressive osteosarcoma cell line, U2OS (Fig. 1, *E* and *F*). Thus, by a systematic analysis of lncRNA expression in cancer patient datasets and then validation in cell lines, we identified an lncRNA, *HMS*, which is upregulated across many cancer types as well as in two established cancer cell lines, where its mechanism of action can be experimentally evaluated. We used two algorithms, coding potential calculator (CPC) and coding–noncoding identifying tool (CNIT), to evaluate the protein-coding potential of *HMS*, and both tools strongly indicated that it is a noncoding RNA.

### HMS depletion causes cell cycle arrest

Subcellular fractionation of U2OS and MG63 cells revealed that *HMS* was primarily localized to the cytoplasm with a minor fraction in the nucleus as has also been reported earlier (42) (Fig. 1*G* and Fig. S1*A*). In order to study the effect of *HMS* depletion, we transduced U2OS cells with lentiviral particles expressing short hairpin RNA (shRNA) against two different region of *HMS* and obtained stable knockdown cells (Fig. 1*H*). To ascertain if *HMS* depletion leads to a G1 accumulation, *HMS* knockdown cells were treated with nocodazole to block the cells in G2/M phase, before evaluating the cell cycle distribution by flow cytometry. Nocodazole treatment reduced the G1 phase population of control cells by blocking the majority of cell population in the G2/M phase; however, the percentage of G1 phase population was significantly higher in *HMS* depleted cells, thus demonstrating a G1 arrest (Fig. 1*I*). Next, we evaluated the rate of DNA synthesis by measuring the incorporation of nucleoside analog, BrdU, using flow cytometry assay. We observed a significant decrease in BrdU incorporation in *HMS* depleted cells as compared with control cells indicating that *HMS* depletion impedes S phase progression (Fig. 1*J*).

### HMS supports aggressive cancer-associated phenotypes

**Figure 2:**
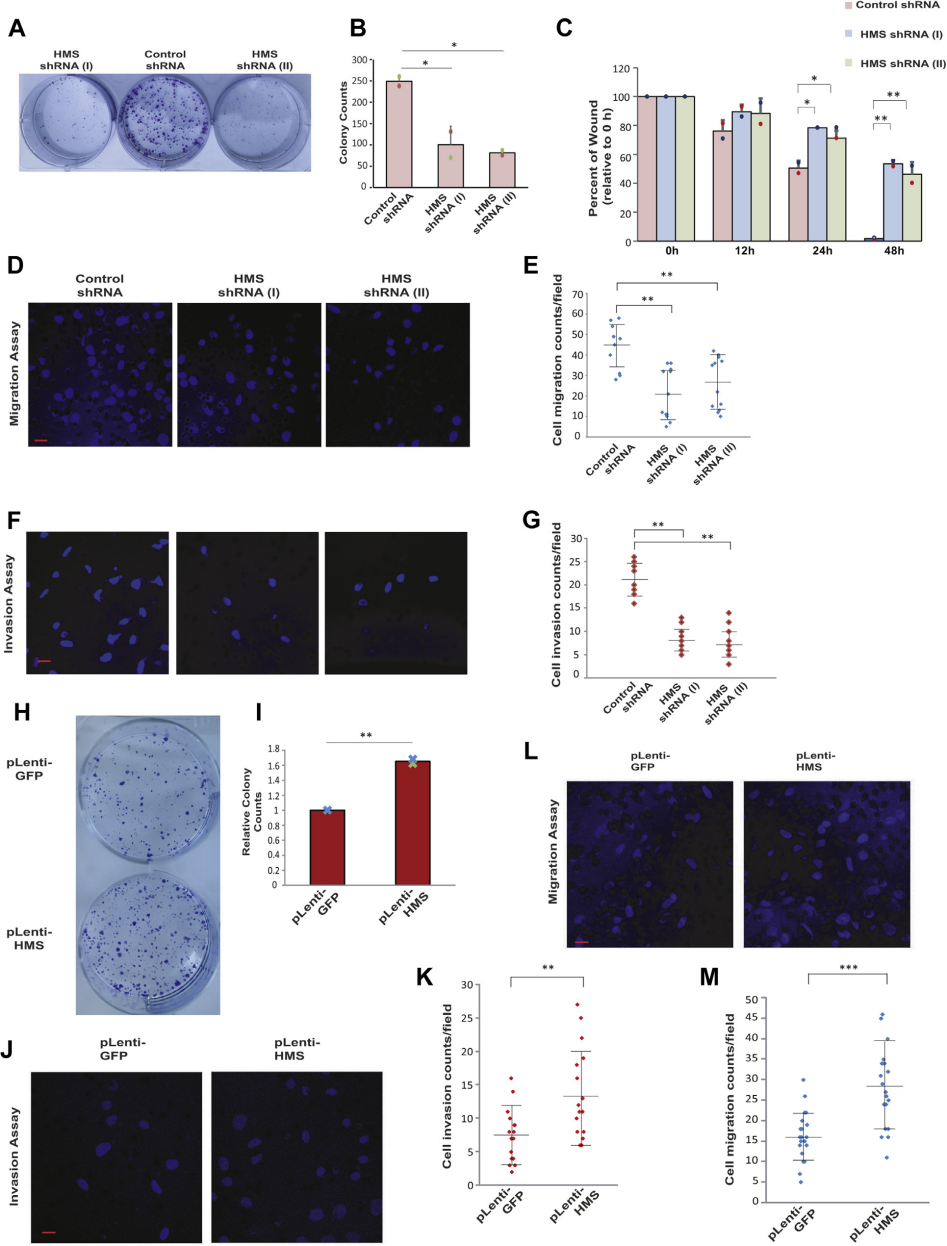
***HMS* supports aggressive cancer-associated phenotypes.***A*, clonogenic assay to evaluate the effect of *HMS* depletion. *HMS*-depleted U2OS cells as described in Figure 1 were allowed to grow for 12 days, stained with crystal violet and the colonies were counted. *B*, quantification of number of colonies observed in part *A*. The data represents mean of two independent experiments ±S.D. Note that both shRNA-I (∗*p* < 0.05, ANOVA/Tukey’s test) and shRNA-II (∗*p* < 0.05, ANOVA/Tukey’s test) significantly reduced the colony forming ability in comparison to control shRNA samples. *C*, effect of *HMS* depletion on wound healing assay: *HMS*-depleted U2OS cells were grown to confluence after which a wound was created using a micropipette tip. The extent of wound healing was monitored at the indicated time points. The data represents mean of two independent experiments ±S.D. Note that in comparison to control shRNA samples, both shRNA-I (∗*p* < 0.05, ANOVA/Tukey’s test) and shRNA-II (∗*p* < 0.05, ANOVA/Tukey’s test) significantly reduced the wound healing capability at 24 h. Similarly, at 48 h shRNA-I (∗∗*p* < 0.01, ANOVA/Tukey’s test) and shRNA-II (∗∗*p* < 0.01, ANOVA/Tukey’s test) significantly reduced the wound healing capability. *D*, *HMS* depletion inhibits cell migration. A representative field showing the DAPI stained nucleus of control or *HMS*-depleted U2OS cells migrating through a microporous membrane counted 24 h after seeding. Scale bar, 20 μm. *E*, quantification of cell migration observed in part *D*. Each point refers to the number of cells in an individual field captured from two independent experiments, whereas *long* and *short horizontal bars* represent the mean and SD respectively of all fields. Note that in comparison to control shRNA samples, both shRNA-I (∗∗*p* < 0.01, ANOVA/Tukey’s test) and shRNA-II (∗∗*p* < 0.01, ANOVA/Tukey’s test) significantly reduced the cell migration. *F*, *HMS* depletion inhibits cell invasion. A representative field showing the DAPI stained nucleus of control or *HMS*-depleted U2OS cells invading through a Matrigel-coated membrane counted 24 h after seeding. Scale bar, 20 μm. *G*, quantification of cell invasion observed in part *F*. Each point refers to the number of cells in an individual field captured from two independent experiments, whereas *long* and *short horizontal bars* represent the mean and SD respectively of all fields. Note that in comparison to control shRNA samples, both shRNA-I (∗∗*p* < 0.01, ANOVA/Tukey’s test) and shRNA-II (∗∗*p* < 0.01, ANOVA/Tukey’s test) significantly reduced the cell invasion. *H*, clonogenic assay to evaluate the effect of *HMS* expression on cell proliferation. U2OS cells were transduced with lentiviral particles produced by transfection of pLenti-HMS or pLenti-GFP vector in HEK293T cells, followed by puromycin selection and generation of stable cells. U2OS cells stably expressing GFP or *HMS* were allowed to grow for 12 days, stained with crystal violet and the colonies were counted. *I*, quantification of number of colonies observed in part *H*. The data represents mean of two independent experiments ±S.D (∗∗*p* < 0.01, Student’s *t* test). *J*, *HMS* expression enhances cell invasion. A representative field showing the DAPI stained nucleus of U2OS cells, stably expressing GFP or *HMS*, as described in part *H*, invading through a Matrigel-coated membrane counted 24 h after seeding. Scale bar, 20 μm. *K*, quantification of cell invasion observed in part *J*. Each point refers to the number of cells in an individual field captured from two independent experiments, whereas *long* and *short horizontal bars* represent the mean and SD respectively (∗∗*p* < 0.01, Student’s *t* test). *L*, *HMS* expression enhances cell migration. A representative field showing the DAPI stained nucleus of U2OS cells, stably expressing GFP or *HMS*, as described in part *H*, migrating through a microporous membrane counted 24 h after seeding. Scale bar, 20 μm. *M*, quantification of cell migration observed in part *L.* Each point refers to the number of cells in an individual field captured from two independent experiments, whereas *long* and *short horizontal bars* represent the mean and SD respectively (∗∗∗*p* < 0.001, Student’s *t* test). Individual data points have been shown for all charts.

To verify the functional significance of *HMS*, we investigated the effect of *HMS* depletion on various tumor-associated phenotypes in U2OS cells. We determined the clonogenic ability after transducing U2OS cells with lentiviral particles expressing shRNA against *HMS* followed by crystal violet staining and colony counting after 12 days. The depletion of *HMS* led to a marked reduction in the colony forming ability of U2OS cells (Fig. 2, *A* and *B*). We further performed wound healing assay by scratching a wound in the monolayer of confluent *HMS* depleted or control cells and then monitoring the size of wound for 48 h. We found that control cells displayed an absolute healing of the wound within 48 h, while in *HMS* depleted cells approximately two-third of the damaged region did not heal in the same duration (Fig. 2*C* and Fig. S1*B*). We utilized *in vitro trans*-well migration and invasion assays to assess the effects of *HMS* on cell migration and invasion ability. We observed that *HMS* depletion inhibited the cell migration ability of U2OS cells (Fig. 2, *D* and *E*). Furthermore, the invasion of U2OS cells through a Matrigel-coated synthetic membrane was significantly suppressed upon *HMS* depletion (Fig. 2, *F* and *J*). Overall, we established that the depletion of *HMS* restricts the invasive phenotypes of U2OS cells.

Next, we tested the effect of ectopic *HMS* expression on the oncogenic phenotypes of U2OS cells. To evaluate the effect of *HMS* on cell proliferation, we performed a clonogenic assay where we observed that colony formation ability was moderately increased upon *HMS* overexpression, which is consistent with higher expression of *HMS* in aggressively proliferating cancer cell lines (Fig. 2, *H* and *I*). Next, we assayed the effect of ectopic *HMS* expression on the ability of U2OS cells to invade the Matrigel-coated membrane where we observed that the cell invasion ability was increased (Fig. 2, *J* and *K*). Moreover, *HMS* overexpression led to an increase in the cell migration ability (Fig. 2, *L* and *M*). Thus, ectopic expression of *HMS* enhances the tumor-related phenotypes of U2OS cells.

### HOXC10 mRNA is downregulated in the absence of HMS

**Figure 3:**
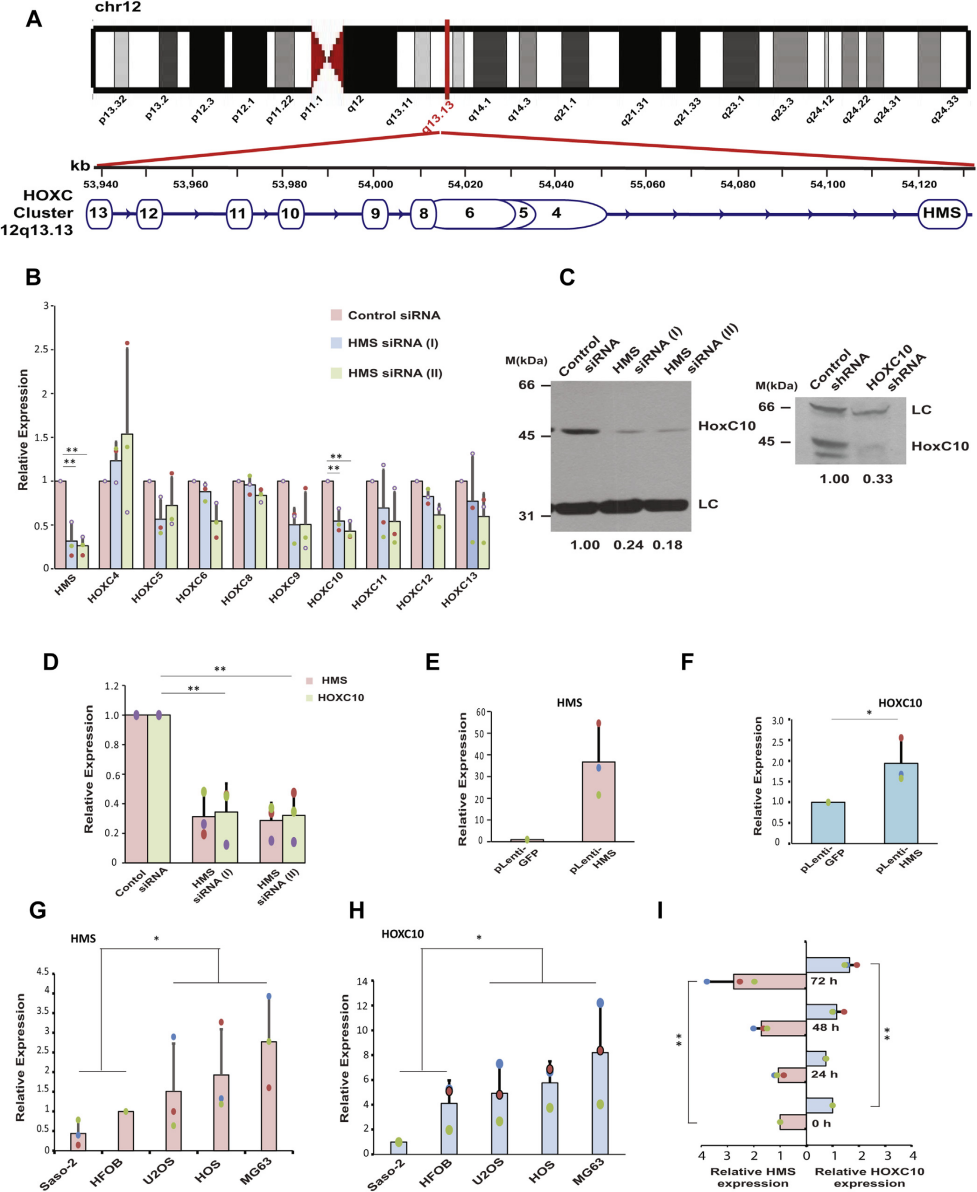
***HOXC10* gene, present 135 kb upstream of *HMS* locus, is downregulated in the absence of *HMS*.***A*, G-banded ideogram representing human chromosome 12, showing the cytogentic location of HOXC cluster and *HMS* gene at the 12q13.13 band. The genomic coordinates shown in kilobases (kb) are as per Genome Reference Consortium Human Build 38 patch release 13 (GRCh38.p13). The HOXC cluster constitutes of nine genes, namely *HOXC13*, *HOXC12*, *HOXC11*, *HOXC10*, *HOXC9*, *HOXC8*, *HOXC6*, *HOXC5*, and *HOXC4*. According to this assembly, the locus of *HOXC10* gene is from 53985146 to 53990279 while the locus of *HMS* gene is from 54126071 to 54132843. The HOX Cluster as well as the *HMS* gene is expressed in the same direction from the “+” strand of chromosome 12, as depicted by *arrows*. *B*, expression of *HOXC10* gene is reduced after *HMS* depletion. U2OS cells were transfected on three consecutive days with one of the siRNAs: control *GL2*, *HMS* siRNA (1), or *HMS* siRNA (2), and the level of genes in the HOXC cluster was quantified by individual quantitative real-time PCR. The *bar graph* indicates the levels of individual HOXC genes in *HMS* depleted samples relative to the control *GL2* sample. *GAPDH* was used as the endogenous control for normalization of HOXC gene expression in different samples. The data represents mean of three independent experiments ±S.D. *HMS* and *HOXC10* transcript levels were significantly reduced after transfection of shRNA (∗∗*p* < 0.01; ANOVA/Tukey’s test). *C*, downregulation of HOXC10 protein after *HMS* depletion. U2OS cells were transfected on three consecutive days with either control or *HMS* siRNA and the level of HOXC10 protein was determined by immunoblotting with anti-HOXC10 antibody. LC, loading control, a nonspecific band that displays equal protein load in each lane. The numbers indicate the level of HOXC10 protein relative to control siRNA transfected cells. *C*, *right panel*, immunoblotting of control shRNA or *HOXC10* shRNA transfected U2OS cell lysate confirms the specificity of anti-HOXC10 antibody. The levels of endogenous *HMS* and *HOXC10* mRNA in the same samples, as determined by quantitative real-time PCR, are shown in part *D*. The data represents mean of three independent experiments ±S.D. (∗∗*p* < 0.01; ANOVA/Tukey’s test). *E*, expression of *HOXC10* gene is moderately increased after overexpression of *HMS*. U2OS cells were transduced with lentiviral particles produced by transfection of pLenti-HMS or pLenti-GFP vector in HEK293T cells, followed by puromycin selection and generation of stable cells. The cells were harvested 48 h after transduction followed by determination of *HMS* and *HOXC10* mRNA levels by quantitative real-time PCR and shown in part *E* and *F*, respectively. The data has been represented as the mean ± SD of three independent experiments (∗*p* < 0.05, Student’s *t* test). *G* and *H*, relative expression of *HMS* and *HOXC10* expression in different OS cell lines with respect to hFOB1.19, a human normal osteoblastic cell line. The expression of *HMS* (part *G*) in both nonaggressive cell lines, hFOB1.19 and Saos-2, is significantly different from the aggressive OS cell lines including U2OS, HOS, and MG63. The data has been represented as the mean ± SD of three independent experiments (∗*p* < 0.05, Student’s *t* test). The levels of *HOXC10* shown in part *H* are significantly different between these two groups of cell lines. The data has been represented as the mean ± SD of three independent experiments (∗*p* < 0.05, Student’s *t* test). *I*, relative expression of *HMS* and *HOXC10* in U2OS cells treated with 200 nM doxorubicin at the indicated time points. The levels of *HMS* and *HOXC10* have been expressed w.r.t. to untreated cells. *GAPDH* was used as the endogenous control for normalization of *HMS* and *HOXC10* expression in different samples. Note that the expression of *HOXC10* increases concurrently with *HMS*. The data represents mean of three independent experiments ±S.D. Note that expression of both *HOXC10* (∗∗*p* < 0.01, ANOVA/Tukey’s test) and *HMS* (∗∗*p* < 0.01, ANOVA/Tukey’s test) was significantly increased at 72 h after doxorubicin treatment. Individual data points have been shown for all charts.

It is known that HOX locus-encoded lncRNAs regulate the expression of other HOX coding genes and since HOXC cluster lies 70 kb upstream at the closest point from the *HMS* locus, we were interested in discerning the role of *HMS* in the regulation of HOXC cluster (Fig. 3*A*) (30, 31). In order to discern the effect of *HMS* on HOXC cluster, we transfected U2OS cells with control or *HMS* siRNAs and evaluated the level of all the genes in the HOXC cluster. We observed that there was no clear pattern in response to *HMS* depletion and the individual HOXC cluster genes responded differently (Fig. 3*B*). However, the expression of *HOXC10* was decreased after depletion of *HMS* with two different siRNAs, confirming that the expression *HOXC10* was dependent on *HMS*. Next, we assayed the levels of HOXC10 protein, which was significantly downregulated after *HMS* depletion (Fig. 3, *C* and *D*). *HMS* is required for the maintenance of *HOXC10* expression so, we next tested the effect of *HMS* overexpression on *HOXC10*. We observed that the expression of *HOXC10* gene was moderately increased after overexpression of *HMS* (Fig. 3, *E* and *F*). Thus, we establish that *HMS* maintains the levels of the *HOXC10* oncogene.

Having identified *HOXC10* as a target of *HMS*, we assayed whether there is a similar expression pattern of *HOXC10* and *HMS* in a panel of OS cells lines displaying varying degrees of aggressive growth phenotypes. We used five different cell lines: (1) hFOB1.19, a human normal osteoblastic cell; (2) Saos-2, a human primary osteogenic sarcoma that displays moderate cancer-related phenotypes such as colony forming ability, proliferation capacity as well as invasive and migratory potential; (3) MG-63, an osteosarcoma cell line, displaying a high proliferation rate and clonogenic ability under anchorage-independent conditions; (4) HOS, a highly tumorigenic osteosarcoma cell line, displaying high invasion and migration potential as well as high proliferation and clonogenic ability; (5) U2OS, an osteosarcoma cell line, displaying high invasion and migration potential as well as high proliferation rate (43, 44). We noted that the expression of *HMS* was significantly higher in aggressive cell lines, U2OS, HOS, and MG63, in comparison to the less aggressive cell line hFOB1.19 and Saos-2 (Fig. 3*G*). Evaluation of *HOXC10* mRNA levels revealed that it is expressed at significantly higher levels in cell lines where *HMS* transcript levels are high, *i.e.*, U2OS, HOS, and MG63 in comparison to hFOB1.19 and Saos-2 cell lines (Fig. 3*H*). Thus, it seems that increased levels of *HOXC10* in transformed cells coincide with the higher levels of *HMS*, supporting the view that *HMS* may be sustaining *HOXC10* levels during oncogenic transformation. It has been previously reported that exposure to doxorubicin induces *HOXC10*, wherein it increases DNA damage repair by homologous recombination thereby, promoting survival of cells (45). We assayed the expression of *HMS* after treatment with doxorubicin and observed that *HMS* displays a pattern of increase, which is similar to *HOXC10*, alluding to an interlinked expression of *HMS* and *HOXC10* (Fig. 3*I*).

### HMS effect on HOXC10 occurs through the 3′UTR of HOXC10

**Figure 4:**
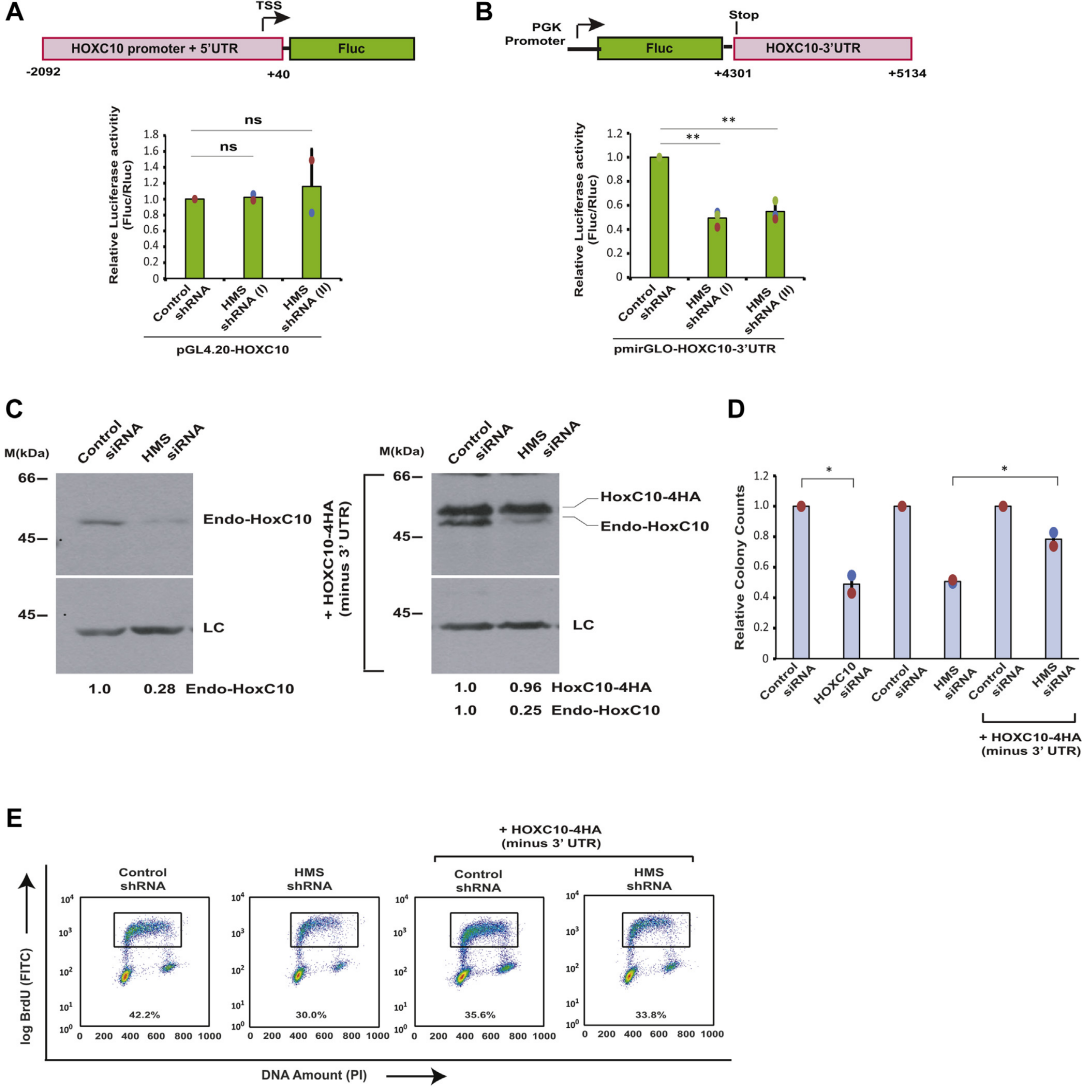
***HMS* effect on *HOXC10* occurs through the 3′UTR of *HOXC10* mRNA.***A*, *HMS* depletion does not affect the *HOXC10* promoter activity. Schematic representation of the reporter plasmid containing the human *HOXC10* upstream region: The *HOXC10* promoter and 5′UTR region spanning −2092 bp to +40 bp with respect to transcriptional start site (TSS) were used to drive expression of the firefly luciferase gene (Fluc) in promoterless pGL4.20 vector (Promega). The pGL4.20 vector containing *HOXC10* promoter was transfected into control or *HMS*-depleted U2OS cells together with a renilla luciferase (pRL-TK) reporter vector and both luciferase activities were measured after 24 h. The relative luciferase activity in each sample is expressed as a ratio of firefly to renilla luminescence. The data represents mean of two independent experiments ±S.D. Note that both shRNA-I (ns, *p* = 0.899, ANOVA/Tukey’s test) and shRNA-II (ns, *p* = 0.830, ANOVA/Tukey’s test) do not significantly alter the luciferase activity in comparison to control shRNA samples. *B*, *HMS* depletion downregulates the *HOXC10* 3′UTR-fused reporter luciferase activity. An illustration of the reporter plasmid containing the human *HOXC10* 3′UTR region: The *HOXC10* 3′UTR region spanning +4301 bp to +5134 bp with respect to TSS was cloned downstream of firefly luciferase ORF under the control of PGK promoter in pmirGLO Dual-Luciferase vector (Promega). The pmirGLO vector containing *HOXC10* 3′UTR was transfected into control or *HMS*-depleted U2OS cells and 24 h later the cells were harvested and luciferase activity was measured. The relative luciferase activity in each sample was expressed as a ratio of firefly to renilla luminescence. The data represents mean of three independent experiments ±S.D. Note that both shRNA-I (∗∗*p* < 0.01, ANOVA/Tukey’s test) and shRNA-II (∗∗*p* < 0.01, ANOVA/Tukey’s test) significantly reduced the luciferase activity in comparison to control shRNA samples. *C*, *HOXC10* expressed without the 3′UTR is impervious to *HMS* depletion. Stable U2OS cells expressing either a control protein (*left panel*) or HOXC10-4HA (without the 3′UTR) (*right panel*) were transfected on three consecutive days with either control *GL2* or *HMS* siRNA. Immunoblotting with α-HOXC10 antibody demonstrates downregulation of endogenous HOXC10 in control stable cells after *HMS* depletion (*left panel*) while exogenous HOXC10-4HA remains stable after *HMS* depletion (*right panel*). *D*, U2OS cells stably expressing a control protein or HOXC10-4HA were transfected with either control *GL2*, *HMS*, or *HOXC10* siRNA, as indicated and the cells were allowed to grow for 12 days and stained with crystal violet after which the colonies were counted. The relative colony counts are normalized to control siRNA for each treatment. The data has been represented as the mean ± SD of two independent experiments. *HOXC10* depletion decreases the colony counts (∗*p* < 0.05, Student’s *t* test). Note that the colony forming ability is moderately but significantly different after HOXC10-4HA expression in *HMS* depleted cells (∗*p* < 0.05, Student’s *t* test). *E*, *HOXC10* expression reverses the *HMS* depletion-induced S phase suppression. U2OS cells stably expressing a control or *HMS* shRNA were transduced with retroviral particles expressing either control protein or HOXC10-4HA, as indicated and pulsed with BrdU followed by flow cytometry to display BrdU incorporation. Individual data points have been shown for all charts.

The most widely reported mechanism of gene regulation by lncRNAs is by modulating the promoter activity of the target genes. In order to test whether *HMS* alters the promoter activity of *HOXC10*, we tested the effect of *HMS* depletion on the activity of luciferase gene driven by the *HOXC10* promoter and 5′UTR region spanning −2092 bp to +40 bp with respect to transcriptional start site (TSS). We did not observe any significant change in the *HOXC10* promoter activity after depletion of *HMS* by two different shRNAs, demonstrating that the effect of *HMS* is not mediated *via* the *HOXC10* promoter (Fig. 4*A*). LncRNAs are known to mediate posttranscriptional gene regulation by modulating the stability of the target RNA (13, 14). Therefore, we assayed whether expression of genes fused to *HOXC10* 3′UTR is altered by modulating the *HMS* levels: The *HOXC10* 3′UTR region was cloned downstream of firefly luciferase, transfected into *HMS*-depleted cells, and the luciferase activity was measured (Fig. 4*B*). We observed that depletion of *HMS* with either of the two shRNAs led to a significant decrease in luciferase activity, demonstrating that *HMS* depletion inhibits the expression of genes fused to *HOXC10* 3′UTR.

We next wanted to determine if the destabilization of endogenous *HOXC10* in the absence of *HMS* involves the 3′UTR of *HOXC10*. We generated stable cells expressing HA-tagged coding sequence of *HOXC10* lacking its 3′UTR, depleted *HMS* by siRNA, and evaluated the stability of HOXC10-4HA protein. We found that while the endogenous HOXC10 protein was downregulated upon *HMS* depletion, the exogenously expressed HOXC10-4HA protein remained unaffected (Fig. 4*C*). This suggests that *HMS* mediates stabilization of oncogene *HOXC10 via* its 3′UTR. We have shown that *HMS* depletion leads to suppression of the cancer-related phenotypes, and thus, next we wanted to establish if this observed suppression is due to the downregulation of *HOXC10*. We transfected HOXC10-4HA expressing stable cells with *HMS* siRNA, which only depletes the endogenous HOXC10 protein without affecting the exogenous HOXC10-4HA, and these cells were then evaluated for their colony forming ability. We observed that the colony forming ability was significantly suppressed upon *HMS* or *HOXC10* depletion but in HOXC10-4HA expressing cells, there was a partial rescue demonstrating that the effect of *HMS* depletion on colony forming ability was due to downregulation of *HOXC10* (Fig. 4*D*). It is likely that *HMS* has other target genes as well and unless there is complementation of all target genes, complete rescue is unlikely to be observed. Similarly, we observed that the *HMS* depletion-induced inhibition of DNA synthesis is rescued after HOXC10-4HA overexpression (Fig. 4*E*). Thus, we conclude that in the absence of *HMS*, *HOXC10* 3′UTR is destabilized leading to decreased levels of HOXC10 protein, which leads to a decrease in the cancer-related phenotypes of U2OS cells.

### RNA-binding proteins, HuR stabilizes the HOXC10 mRNA transcript *via* its 3′UTR

**Figure 5:**
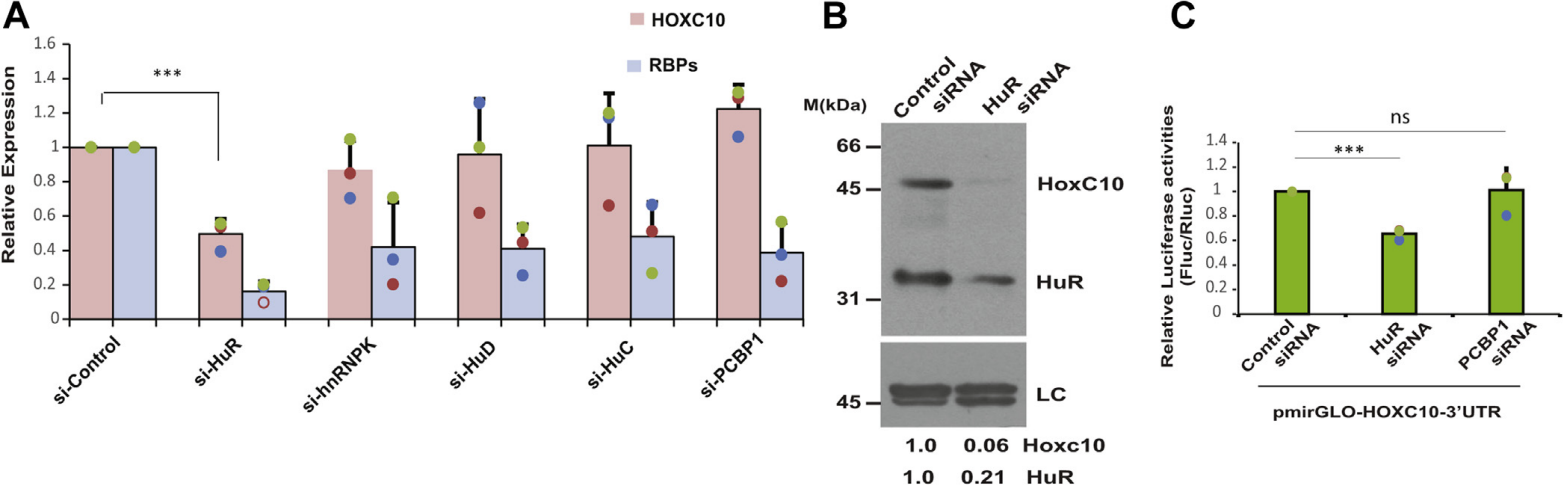
***HOXC10* 3′UTR is destabilized in the absence of ELAV family protein, HuR.***A*, depletion of HuR downregulates *HOXC10* mRNA. U2OS cells were transfected on three consecutive days with siRNA targeting individual RBPs (HuR; hnRNPK; HuD; HuC; PCBP1). The *bar graph* indicates the levels of specific RBPs and *HOXC10* mRNA quantified by quantitative real-time PCR in different samples relative to control siRNA samples. *GAPDH* was used as the endogenous control for normalization of *HOXC10* gene expression in different samples. The data has been represented as the mean ± SD of three independent experiments. Note that *HOXC10* mRNA levels are significantly reduced after transfection of HuR siRNA (∗∗∗*p* < 0.0001, Student’s *t* test). *B*, downregulation of HOXC10 protein after HuR depletion. U2OS cells were transfected on three consecutive days with either control or HuR siRNA and levels of *HOXC10* and HuR protein were determined by immunoblotting. LC, loading control, a nonspecific band that displays equal protein load in different lanes. The numbers indicate the levels of *HOXC10* and HuR proteins relative to control siRNA transfected cells. *C*, HuR depletion downregulates the *HOXC10* 3′UTR-fused reporter luciferase activity. PmirGLO vector with the *HOXC10* 3′UTR cloned downstream of firefly luciferase ORF was transfected into U2OS cells depleted for HuR or PCBP1 and 24 h later luciferase activity was measured. The relative luciferase activity in each sample was expressed as a ratio of firefly to renilla luminescence. The data represents mean of three independent experiments ±S.D. Note that HuR siRNA-I significantly reduced the luciferase activity in comparison to control shRNA samples (∗∗∗*p* < 0.001, Student’s *t* test) while PCBP1 siRNA did not significantly alter the luciferase activity in comparison to control shRNA samples (ns, *p* = 0.9164, Student’s *t* test). Individual data points have been shown for all charts.

The above results establish that *HMS* stabilizes the *HOXC10* mRNA transcript through its 3′UTR, and thus, we next wanted to discern the mechanism by which *HMS* stabilizes the *HOXC10* mRNA. It is known that lncRNAs recruit RBPs to 3′UTR of mRNA transcripts in order to modulate the RNA stability (13, 14). Therefore, we evaluated the contribution of specific RBPs that have putative binding sites in the *HOXC10* 3′UTR and are known to stabilize mRNA transcripts. This includes (1) ELAV family proteins, HuR, HuC, and HuD, that prevent the destabilizing RBPs from binding to mRNA transcript; (2) PCBP1, which binds to the 3′UTR of p27 mRNA stabilizing it; and (3) hnRNP-K, which is known to stabilize the *CDK6* mRNA in association with lncRNA *MYU* (14, 20, 46). To evaluate the effect of HuR, HuC, HuD, hnRNP-K, and PCBP1 on *HOXC10* mRNA stability, we transfected the cells with respective siRNAs, which decreased the targeted RBPs to varying levels (Fig. 5*A*). We observed a significant decrease in the endogenous levels of *HOXC10* mRNA after depletion of HuR, while depletion of HuC, HuD, hnRNP-K, or PCBP1 did not significantly decrease *HOXC10* mRNA levels. The HuR depletion-induced *HOXC10* mRNA downregulation also led to a significant decrease in HOXC10 protein (Fig. 5*B*).

In the earlier part of this paper, we have shown that the stabilization of *HOXC10* by lncRNA *HMS* is mediated *via* 3′UTR of *HOXC10* mRNA so, next we wanted to evaluate if the effect of RBP HuR on *HOXC10* stability was also through *HOXC10* 3′UTR. We utilized a reporter vector, where *HOXC10* 3′UTR was cloned downstream of firefly luciferase ORF and transfected it into U2OS cells depleted for HuR or PCBP1. We observed that HuR depletion decreased the luciferase activity to around 60% of that of the control cells, while PCBP1 depletion did not decrease the luciferase activity. This proves that the HuR depletion-induced downregulation of *HOXC10* mRNA is due to the 3′UTR of *HOXC10* (Fig. 5*C*). Thus, we have successfully identified the RBP, HuR, which mediates the stabilization of *HOXC10* RNA through its 3′UTR.

### HMS recruits HuR to the HOXC10 3′UTR to stabilize it

**Figure 6:**
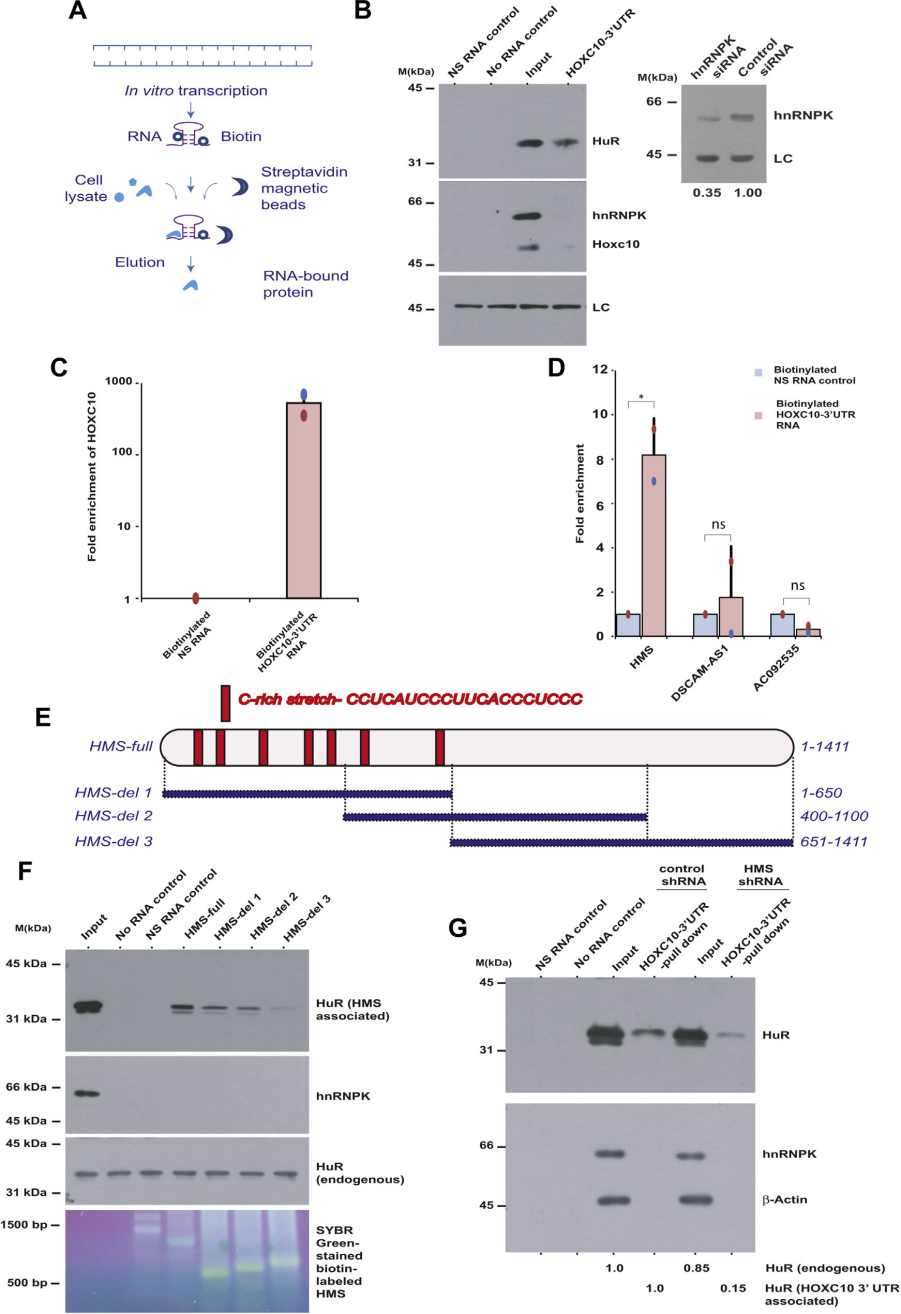
**HuR physically interacts with the cytosine-rich stretch of *HMS* to stabilize *HOXC10* 3′UTR.***A*, experimental design of RNA pull-down assay for identifying RNA-associated cellular proteins. RNA sequence was cloned in pCDNA3 plasmid downstream of the T7 RNA polymerase promoter followed by *in vitro* transcription in the presence of biotin-14-CTP to synthesize biotin-labeled RNA fragments. For RNA pull-down assay, the cell lysate was incubated with biotin-labeled RNA and streptavidin magnetic beads followed by washing, elution, and immunoblotting to detect the RNA-associated proteins. *B*, HuR physically associates with *HOXC10* 3′UTR. As per the protocol described in part *A*, *in vitro* synthesized biotinylated *HOXC10* 3′UTR RNA was incubated with U2OS cell lysates to identify proteins associated with biotinylated *HOXC10* 3′UTR. NS control is a nonspecific commercial biotin-labeled *in vitro* transcribed RNA while “no-RNA” serves as the negative control for nonspecific binding. Input lane shows 10% of total cell lysate used for the pull-down assay. LC displays that equal cell lysate was loaded in each assay. Note that hnRNP-K does not bind to *HOXC10* 3′UTR. *B*, *right panel*, immunoblotting of control *GL2* or hnRNP-K siRNA transfected U2OS cell lysate confirms the specificity of anti-hnRNP-K antibody. *C* and *D*, RNA pull-down assay to identify the lncRNAs associated with biotinylated-*HOXC10* 3′UTR. Biotinylated-*HOXC10* 3′UTR was used for the precipitation of RNA from U2OS cell lysates and qRT-PCR was performed to identify the associated lncRNAs, *HMS*, *DSCAM-AS1*, and *AC092535*. Part *C* displays that biotinylated *HOXC10* 3′UTR bound effectively to the streptavidin beads, while Part *D* shows that *HMS* was specifically enriched with *HOXC10* 3′UTR bound beads. The data represents mean of two independent experiments ±S.D. Note that *HMS* associates with *HOXC10* 3′UTR in comparison to nonspecific RNA control (∗*p* < 0.05, Student’s *t* test) while *AC092535* and *DSCAM-AS1* (ns, *p* = 0.689, Student’s *t* test) do not significantly associate with *HOXC10* 3′UTR. *E*, schematic representation of *HMS* displaying its seven cytosine-rich stretches. The sequence of one of the cytosine-rich stretches has been shown at the top. The illustration shows the full-length and deletion fragments of *HMS* used for the precipitation of RNA-binding proteins from U2OS cell lysates. *F*, HuR physically associates with cytosine-rich stretches of *HMS*. Full-length *HMS* or its deletion fragments, as indicated in part *E*, were cloned in pCDNA3 downstream of the T7 RNA polymerase promoter followed by *in vitro* transcription in the presence of biotin-14-CTP to synthesize biotin-labeled RNA fragments. For RNA pull-down assay 1 μg of biotinylated full-length *HMS* or deletion fragments, as indicated, were used for the precipitation of HuR from U2OS cells and immunoblotting was performed to identify the associated HuR protein (*top panel*). SYBR-Green stained denaturing agarose gel displays that equal amounts of biotin-labeled full length *HMS* or deletion fragments were used for RNA pull-down assay (*third panel*). *G*, the physical association between HuR and *HOXC10* 3′UTR is disrupted in the absence of *HMS*. As described in part *B*, biotinylated *HOXC10* 3′UTR was used for the precipitation of HuR from U2OS cells transfected with either *HMS* or control *GL2* siRNA. The input lane in *GL2* and *HMS* siRNA samples shows that the endogenous HuR protein levels are not significantly decreased after *HMS* RNAi (0.85 fold in comparison to *GL2* samples). However, the HuR associated with *HOXC10* 3′UTR is significantly decreased after *HMS* depletion (0.15 fold in comparison to *GL2* samples). The amount of HuR associated with *HOXC10* 3′UTR was normalized to the levels of endogenous HuR in *GL2* and *HMS* siRNA samples. Individual data points have been shown for all charts.

The results above show that the *HOXC10* 3′UTR stabilization requires both HuR and *HMS*. We now wanted to ascertain the mechanism by which HuR and *HMS* stabilize the *HOXC10* 3′UTR and thus, we assayed the physical interaction between *HOXC10* 3′UTR, HuR, and *HMS*. We used an RNA pull-down assay where biotin-labeled *HOXC10* 3′UTR was synthesized *in vitro*, bound to streptavidin beads followed by incubation with cell lysate to detect the associated proteins (Fig. 6*A*). Immunoblotting with anti-HuR antibody revealed that HuR physically associates with *HOXC10* 3′UTR while another RBP, hnRNPK, does not (Fig. 6*B*). To identify the lncRNAs that physically interact with *HOXC10* 3′UTR, biotin-labeled *HOXC10* 3′UTR bound to streptavidin beads was incubated with cell lysate followed by qRT-PCR to identify associated lncRNAs (Fig. 6*C*). We observed that while *HMS* was specifically enriched with *HOXC10* 3′UTR bound beads, other lncRNAs, *DSCAM-AS1* and *AC092535*, did not show any affinity for *HOXC10* 3′UTR, demonstrating that *HOXC10* 3′UTR specifically associates with *HMS* (Fig. 6*D*).

Next, we ascertained the physical association between HuR and *HMS* and for that biotin-labeled *HMS* was synthesized *in vitro* and incubated with cell lysate to detect the associated proteins. We observed that HuR physically associates with *HMS* while hnRNPK does not (Fig. 6*F*, lane 4). In order to identify the region of *HMS* that physically associates with HuR, we synthesized three deletion fragments of *HMS in vitro* (*HMS*-del1, -del2, -del3) and assayed for HuR association with deletion mutants of *HMS* (Fig. 6*E*). HuR is known to bind to AU-rich elements (AREs; AUUUA pentamers and/or UUAUUUA(U/A)(U/A) nonamers) that are found in the 3′ UTR of many mRNAs (18). However, *HMS* does not have a canonical AUUUA motif, which is not exceptional as HuR also binds to mRNAs that lack the canonical ARE motif. These other non-ARE elements include a U-rich polypyrimidine tract with a C(U)nC motif, a C-rich *cis*-element, and polypyrimidine tracts located between the splice site and the branch point of pre-mRNAs (21, 22, 47). We observed that *HMS*-del1 and *HMS*-del2 retained physical association with HuR but *HMS*-del3 did not bind to HuR (Fig. 6*F*, lanes 5–7). Closer examination of the sequence of *HMS* revealed that it has seven cytosine-rich stretches whose density is least in the *HMS*-del3 fragment. Thus, our results suggest that HuR physically associates with the cytosine-rich stretches of *HMS*. None of the fragments showed any physical association with hnRNPK, verifying the specificity of *HMS* association with HuR.

Next, we wanted to ascertain if the reason for *HMS* depletion-triggered destabilization of *HOXC10* 3′UTR is the dissociation of HuR-*HOXC10* 3′UTR complex. We assayed for physical association of *in vitro* synthesized *HOXC10* 3′UTR with endogenous HuR from cell lysates obtained from either *HMS* or control depleted cells. Though, there were equivalent levels of *HOXC10* 3′UTR and HuR in each sample, HuR-*HOXC10* 3′UTR association was disrupted in *HMS* depleted samples (Fig. 6*G*, lane 6). This implies that *HMS* is essential for the recruitment of HuR to *HOXC10* 3′UTR for its stabilization. Thus, we conclude that the process of stabilization of *HOXC10* 3′UTR involves physical association between *HMS* and HuR and recruitment to the *HOXC10* 3′UTR.

### Correlation between HOXC10 and HMS expression in human cancer samples

**Figure 7:**
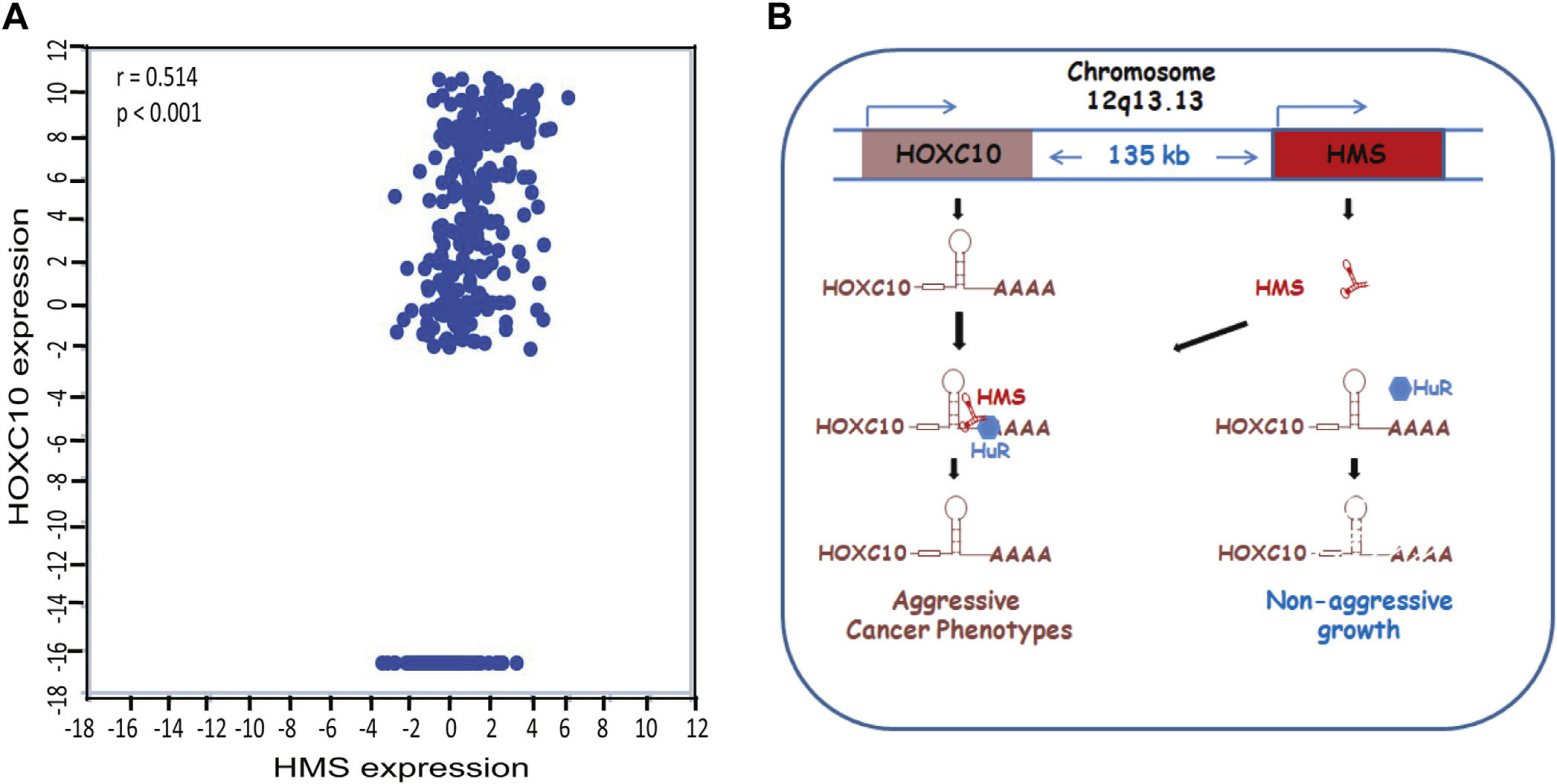
**Model depicting *HMS*-mediated stabilization of *HOXC10* by recruitment of RNA stabilizing protein, HuR.***A*, correlation analysis between the expression of *HMS* and *HOXC10* in the LUAD samples of the TCGA database. Spearman rank correlation was used to assess relationships between lncRNA and mRNA expression, which showed a positive correlation coefficient, “r” >0.5 with a *p*-value <0.001 (Student’s *t* test). *B*, The *HOXC10* gene is located 135 kb upstream of the *HMS* gene locus and both are expressed in the same direction from the “+” strand of chromosome 12, as depicted by *arrows*. At low levels of *HMS*, the RNA stabilizing protein HuR is not recruited to the 3′ untranslated region of *HOXC10* mRNA resulting in its destabilization (depicted on the *right side* of the illustration). In the presence of *HMS*, HuR is recruited to the *HOXC10* 3′UTR stabilizing its mRNA and thus maintaining the oncogenic properties of cancer cells (*left side* of the illustration). The Genotype-Tissue Expression (GTEx) data reveals that in most of the tissues a high *HOXC10* expression is accompanied by high levels of *HMS* expression.

We began this study by analyzing the transcriptional profiles of TCGA samples, wherein we identified dysregulated lncRNAs including *HMS*, and thereafter we demonstrated that *HMS* stabilizes the mRNA of oncogene *HOXC10*. If this mechanism is functional in human cancer tissues, then coherence in *HOXC10* and *HMS* levels is likely to be observed. We analyzed tumor samples of the TCGA-LUAD database and observed a positive correlation between the expression of *HMS* and *HOXC10* (Fig. 7*A*). A high correlation coefficient (r > 0.5) suggests that *HMS* could be one of the factors sustaining the expression of *HOCX10* in tumor tissues.

## Discussion

The results of this study lead to many significant implications. First, the rescue of oncogenic phenotype upon *HOXC10* expression in *HMS* depleted cells proves that *HMS* and *HOXC10* work together to maintain oncogenesis. The positive correlation between the expression of *HMS* and *HOXC10* was conserved not only in established cancer cell lines but also in human tumor tissues making a strong case that *HMS* sustains *HOXC10* levels during oncogenic transformation. Second, the high levels of both *HMS* and *HOXC10* observed in cancer patient samples are associated with low survival probability, which further emphasizes that *HMS* and *HOXC10* both contribute to human oncogenic pathology (Fig. S1, *C* and *D*). Third, the genotype-tissue expression (GTEx) data shows that *HMS* levels are high in most of the tissues where *HOXC10* is high, which suggests that *HMS* may regulate *HOXC10* levels even in normal adult tissues. Next, since *HOXC10* plays a vital role in the morphogenesis in multicellular organisms, one of the implications of the present study is that it opens the possibility that lncRNAs such as *HMS* regulate *HOXC10* during mammalian development (48, 49, 50).

Recent studies have reported the role of *HMS* in human oncogenesis: It has been reported that *HMS* is upregulated in cervical cancer tissues, and its depletion suppresses cell invasion, which was reversed by transfecting an miR-133b inhibitor, demonstrating that the oncogenic effect of *HMS* on cell growth was by sponging miR-133b (51). Another study demonstrated that *HMS* sponges another miRNA, miR-503-5p to upregulate cell cycle gene *CDCA4*, establishing an oncogenic role of *HMS* in osteosarcoma (52). A recent report has also linked *HMS* to noncancer diseases such as rheumatoid arthritis, wherein it was shown that *HMS* overexpression enhanced cell proliferation of fibroblast-like synoviocytes by sponging miR-590-5p and activating the MAPK signaling pathway (53). Contrary to the above reports, one group has reported that ectopic expression of *HMS* in colorectal and gastric cancer cells decreased cell viability and colony formation capability and proposed that *HMS* has tumor-suppressive effects on human colorectal tumorigenesis (54, 55). While this could indicate that *HMS* has a cancer-specific role, pleiotropic effects resulting from any experimental alterations have to be ruled out. Nonetheless, the above reports establish that *HMS* regulates cell proliferation in multiple human cancers.

LncRNAs are known to regulate genes that are located close to their own site of transcription, and this led us to focus on HOXC cluster, which lies 70 kb upstream of the *HMS* locus. There are several examples of lncRNAs expressed from or around the four HOX gene clusters (HOX-A, B, C, D) that regulate closely located genes. *HOTTIP*, a lincRNA transcribed from the 5′ tip of the HOXA locus, regulates several 5′ HOXA genes while another lncRNA, *linc-HOXA1*, expressed from around 50 kb upstream of the HOXA gene cluster represses the closest located *HOXA1* gene (31, 56). Though HOXC cluster embedded lncRNAs *HOTAIR* represses transcription at the HOXD locus, little is known about the lncRNA regulation of HOXC cluster. Since HOXC cluster lies close to the *HMS* locus, we were interested in discerning if *HMS* has any role in its regulation. Moreover, many HOXC cluster genes were known to support tumorigenesis, which could be related to the oncogenic activity of *HMS* (48, 49).

In the present study, we screened for dysregulated lncRNAs in the lung adenocarcinoma samples as previous studies have reported that the number of lncRNAs upregulated in lung adenocarcinoma was one of the highest amongst human cancers: 641 out of total 4470 detectable lncRNAs are upregulated by more than twofold in LUAD samples (33). Significantly, more than 82% of lncRNAs that were upregulated in LUAD were also upregulated in more than one cancer type, a feature that distinguishes LUAD from other cancers. Since, our objective was to identify lncRNAs whose dysregulation was conserved across cancer types, we reasoned that LUAD was an appropriate system to delineate dysregulated lncRNAs having tumor-related functions.

*HOCX10* gene is closely located to the *HMS* locus (135 kb distance), which raises the prospect of *HMS* functioning as a *cis*-acting lncRNA, but in the course of this study it became apparent that *HMS* does not activate the *HOXC10* gene by a *cis*-acting mechanism. First, *trans* expression from a lentiviral vector induced *HOXC10*. Next, luciferase activity linked to *HOXC10* 3′UTR located on an exogenous pmirGLO vector was downregulated by *HMS* depletion, again ruling out that *HMS* only functions in *cis*, near its transcription site. Similarly, binding of biotin-labeled *in vitro* transcribed *HOXC10* mRNA with *HMS* proves that the stabilization of *HOXC10* by *HMS* is not by a *cis*-acting mechanism. In summation, in this study we demonstrate that *HMS* recruits the RNA stabilizing protein called HuR to the 3′ untranslated region of oncogene *HOXC10*, thereby stabilizing its levels and maintaining the oncogenic properties of the cancer cells. The most widely reported mechanism of lncRNAs function is regulating gene transcription by epigenetic modification but understanding posttranscriptional mechanisms such as those described in our study would be another step in comprehending the landscape of cancer gene regulation that would aid in therapeutic intervention against cancer.

## Experimental procedures

### Cell culture, cell synchronization, and cloning

U2OS, HOS, MG-63, and Saos-2 (human osteosarcoma cell lines) and HEK293T (human embryonic kidney cells with SV40 large T antigen cell line) were maintained in Dulbecco’s modified eagle’s medium (DMEM) supplemented with 10% fetal bovine serum (FBS) along with 1% of 100 units/ml antibiotic and antimycotic solution at 37 °C in a humidified atmosphere with 5% CO_2_. HFOB1.19 (human fetal osteoblastic cell line) was maintained in 1:1 mixture of Ham’s F12 Medium and DMEM supplemented with 10% FBS and 1% of 100 U/ml of antibiotic and antimycotic solution at 34 °C in a humidified atmosphere containing 5% CO_2_. A549 (adenocarcinomic human alveolar epithelial cell line) cells were maintained in DMEM and BEAS-2B (immortalized but a nontumorigenic lung epithelial cell line) was maintained in 1:1 of F12 and DMEM low-glucose medium. For constructing retroviral vectors expressing 4HA-tagged HOXC10, its cDNA was amplified by PCR and cloned into murine leukemia virus long terminal repeats (LTRs)-driven plasmid, pMX-puro-4HA. HEK293T cells were transfected with pMX-puro-HOXC10-4HA along with helper plasmids expressing the viral VSV-G envelope protein, as well as the Gag and Pol proteins to generate viral particles. To obtain stable cells expressing HOXC10-4HA, U2OS cells were infected with the retroviral particles along with 1 μg/ml of polybrene and selected with 1 μg/ml of puromycin 48 h after the infection. For constructing lentiviral vectors targeting *HMS*, two shRNAs that target the *HMS* at different regions were inserted into AgeI/EcoRI-digested pLKO.1 puro (Addgene). For lentivirus preparation, lentiviral vector pLKO.1 expressing shRNA was cotransfected with packaging vector pMD2.G and envelope vector psPAX2 at a 4:3:1 ratio using Lipofectamine 2000 reagent (Invitrogen) in HEK293T cells. To obtain stable cells expressing shRNA, U2OS cells were infected with the lentiviral particles along with 1 μg/ml polybrene and selected with 1 μg/ml of puromycin 24 h after the infection. For expression of *HMS*, GFP was replaced by full-length *HMS* in lentiviral vector pLenti-GFP. For biotin-labeled RNA pull-down assay, *HMS* or *HOXC10* 3′UTR sequence was cloned in pCDNA3 plasmid downstream of the T7 RNA polymerase promoter.

### Transfection

For RNAi-mediated gene silencing, small inhibitory RNAs (siRNAs) against *GL2*, *HMS*, HuR, hnRNPK, and *PCBP1* were custom synthesized by Dharmacon. Cells were transfected with 80 nM of siRNA using Lipofectamine 2000 reagent (Invitrogen) for three consecutive days. The cells were harvested 24 h after the last transfection for immunoblotting, flow-cytometric analysis, or reverse transcriptase PCR. The siRNA sequences used are as follows:

*GL2*: CGUACGCGGAAUACUUCGA;

*HMS* shRNA (I): AAGAGGTCGAGGGAATGTTGT; *HMS* shRNA (II): TCTGGCCAGGAGGAAGGGCAG;

*HMS* siRNA (I): AAGAGGUCGAGGGAAUGUU; *HMS* siRNA (II): GCUGAGAGCCGCAGUUGCA;

*HOXC10* siRNA: CGGAUAACGAAGCGAAAGA

*PCBP1* siRNA is a pool of four siRNAs: UCGCUAUGAUCAUCGACAA; CAACUUUAUCAUCCGCUAA; CAAAGAGAUCCGCGAGAGU; CUACUCGAUUCAAGGACAA;

HuR siRNA is a pool of four siRNAs: GACAAAAUCUUACAGGUUU; GACAUGUUCUCUCGGUUUG; ACAAAUAACUCGCUCAUGC; GCUCAGAGGUGAUCAAAGA.

HuC siRNA is a pool of four siRNAs: CCUCAACGGCCUCAAAUUA; GCGAACAACCCAAGUCAGA; GCAAGUUGGUUCGGGACAA; UCAAGGUCAUCCGUGAUUU.

HuD siRNA is a pool of four siRNAs: GGUAUGGAUUUGUUAACUA; GGAACUGGGUGGUGCAUCU; CUACGGAACCGAUUACUGU; CAGGGAUGCUAACCUCUAU.

The RT-PCR primers are as follows:

*HMS*: CCTCCCGGAATCGTTTGAGA; TTTGCCGAGCAGATGTGGAT.

*HOXC10*: GGATAACGAAGCGAAAGAGGAG; TCCAGCGTCTGGTGTTTAGT.

*HOXC10*, 3′UTR specific primer: GAATCGTTCCTTCCTTGCTGC; ACACGAACACTAGCCGAACT.

HuR: CCCTCTGGATGGTGGTGAAC; AAGCGGTTGAGAAAACGCAC.

### Luciferase reporter assay

The firefly luciferase-encoding reporter plasmids pGL4.20 [luc2] and pRL-TK were obtained from Promega. The pRL-TK, which encodes renilla luciferase, was used as an internal control for transfection efficiency. The −2092 bp to +40 bp upstream region of *HOXC10* transcription start site was cloned into pGL4.20. Control or *HMS*-depleted U2OS cells were cotransfected with pGL4.20-*HOXC10* and pRL-TK, and 24 h later the cells were lysed and firefly and renilla luciferase luminescence was sequentially measured according to the manufacturer’s protocol. The firefly luciferase activity was normalized to renilla luciferase activity. To construct a 3′ UTR luciferase reporter plasmid, the *HOXC10* 3′UTR region spanning +4301 bp to +5134 bp with respect to TSS was cloned downstream of firefly luciferase ORF under the control of PGK promoter in pmirGLO Dual-Luciferase vector (Promega). For luciferase reporter assay, the pmirGLO vector containing either WT- *HOXC1*0 3′UTR was transfected into *HMS* or control shRNA depleted U2OS cells, and the cells were harvested after 24 h for measurement of luciferase activity using Dual-Luciferase reporter assay kit (Promega).

### Cell cycle analysis and flow cytometry

For cell cycle analysis, the cells were harvested and fixed with 70% ethanol at 4 °C for 1 h. Following fixation, the cells were washed with 1× PBS, and the cell pellet was resuspended in 1× PBS with 0.1% Triton X- 100, 20 mg/ml RNase A, and 70 mg/ml propidium iodide, and then the stained cells were analyzed by flow cytometry. For arresting the cells at G2/M transition, the cells were incubated with nocodazole (100 ng/ml) for 16 h before harvesting and fixation with 70% ethanol. The flow cytometry data was acquired on Becton Dickinson FACS Canto machine using BD FACS Diva software. Cell cycle distribution was evaluated by Dean/Jett/Fox method using the FlowJo software. To study the BrdU (5-bromo-2-deoxyuridine) incorporation, cells were cultured in medium containing 100 μM BrdU (BD Biosciences) for 30 min, prior to harvesting. After fixation, cells were treated with 2 N HCl for 15 to 20 min for denaturing the DNA, followed by a neutralization step of 5 min at room temperature with 0.1 M sodium tetraborate (pH 8.5). Cells were then washed with a blocking solution comprising 3% bovine serum albumin (BSA) in PBS containing 0.1% Triton X-100 followed by incubation with mouse anti-BrdU antibody (dilution 1:10 in blocking solution) conjugated to Fluorescein isothiocyanate (FITC) for 1 h. After antibody staining, cells were washed with 1× PBS, and DNA was stained with propidium iodide and run on FACS machine as previously described.

### RNA extraction and quantitative real-time PCR

Total RNA was extracted from cells using TRIzol reagent (Takara Biosciences) and reverse transcribed into cDNA using Moloney murine leukemia virus reverse transcriptase (Invitrogen). The qRT-PCR reactions were carried out in duplicates in 10 μl volume for the expression analysis. The reaction mixture contained SYBR Select master mix (2×, Takara Biosciences), cDNA template, and forward and reverse gene or lncRNA specific primers (0.1 μM each). Target sequence amplification temperature profile followed was as follows: Initial denaturation for 10 min at 95 °C, followed by 40 cycles of 10 s at 95 °C and amplification for 30 s at annealing temperature of 60 °C. Finally, a melt curve analysis was carried out at a temperature range of 60 to 95 °C for 20 min. The *GAPDH* was used as internal control for lncRNA and mRNA quantification. Results were calculated using ΔΔCt method to determine the fold change in expression between the experimental and control groups.

### Immunoblotting and antibodies

For western blotting, the whole cell lysates from equally confluent cultures were prepared in proportionate volume of laemmli buffer and denatured at 95 °C followed by SDS-PAGE. The gel was transferred onto a nitrocellulose membrane, blocked with 5% milk prepared in 1× TBST. The membrane was then incubated with the appropriate antibody, washed and probed with horseradish-peroxidase-conjugated secondary antibody. Enhanced chemiluminescence was used to visualize the protein bands. Quantity One Software (version 4. 6. 3; Bio-Rad) was utilized to quantitate the levels of specific proteins, which were expressed after normalization with the protein loading control. The following antibodies were used: mouse anti-hnRNPK antibody (ab23644, Abcam), rabbit anti-HuR antibody (ab200342, Abcam) and rabbit anti-HOXC10 antibody (orb539862, Biobryt Biotechnology). Antibodies to detect hnRNPK, HuR, and HOXC10 were validated by immunoblotting with siRNA-transfected cell lysates. Antibodies against loading control proteins Cdk2 (Sc-163) and β-actin (Sc-97778) were obtained from Santa Cruz Biotechnology. Horseradish-peroxidase-linked anti-mouse IgG (Cat no. 7076, Cell Signaling Technology) and horseradish-peroxidase-linked anti-rabbit IgG (Cat no. 7074, Cell Signaling Technology) were used as secondary antibodies in immunoblotting.

### Cell proliferation assays

For MTT cell proliferation assay, 30,000 U2OS cells were seeded in triplicates in 96-well cell culture dishes with 500 μl media per well. The MTT substrate, thiazolyl blue tetrazolium bromide, was added to cells in culture at a final concentration of 0.5 mg/ml and incubated at 37 °C. After 3 to 4 h, the cells were resuspended in 500 μl of dimethyl sulfoxide (DMSO) and shaken for 15 min. The quantity of formazan was measured by recording changes in absorbance at 570 nm and 630 nm (reference wavelength) using a microplate reader (BioTekPowerWave XS). For cell viability count, trypan blue exclusion method was utilized where *HMS*-depleted or control U2OS cells were collected and dissolved in 1 ml of 1× PBS, and 20 μl of cell suspension was stained with an equal volume of 0.4% trypan blue. Viable cells, which excluded trypan blue dye, were counted in quadruplicate using a glass hemocytometer.

### Wound healing and clonogenic assay

*HMS*-depleted or control U2OS cells seeded in 6-well plates with an approximate confluence of 30 to 40% were cultured until confluence. A wound was then created by manually scraping the cell monolayer with a 200 μl pipette tip. The cultures were washed twice with 1× PBS and supplemented with fresh medium. Cell movement into the wound was observed at four preselected time points (0, 12, 24, and 48 h) in eight randomly selected microscopic fields for each condition and time point. Images were captured with a Plan Fluor 10×/0.3 objective of a Nikon TE2000-S inverted microscope on Evolution VF (Media Cybernetics) 12 bit color digital camera using the “Q capture Pro” software. The distance traveled by the cells was determined by measuring the wound width at different time points. For clonogenic assay, *HMS*-depleted or control U2OS cells were counted and 1000 cells were seeded in a 6-well culture dish. After 12 days of incubation, plates were gently washed with 1× PBS and stained with 0.1% crystal violet. Colonies with over 50 cells were manually counted.

### Invasion and migration assays

*HMS*-depleted or control U2OS cells (6 × 10^4^) resuspended in 250 μl serum-free medium were seeded into the upper well of the Matrigel-coated membrane of a transwell chamber (8 μm pore size, Corning) for assaying cell invasion. For migration assays, the cells were seeded into the upper well of millicell hanging cell culture insert of a transwell chamber (8 μm pore size, Millipore). Serum containing medium was added to the lower chamber and cells were incubated at 37 °C for 24 h. Subsequently, cells in the upper chamber were removed and the cells migrating to or invading the bottom of the membrane were fixed with cold methanol and mounted with Vectashield mounting reagent containing DAPI (4′, 6-diamidino-2-phenylindole) that stains the nucleus. Images were captured with a Plan apochromat 40×/1.0 oil objective of a Nikon Eclipse Ti inverted microscope after excitation of DAPI with a violet (405 nm) laser line using a Nikon A1 confocal laser-scanning system. The images were captured using the NIS-Elements imaging software (version 3.22.00) for quantification of cells. Multiple random fields of each membrane were photographed and counted for statistical analysis.

### Biotin labeling and pull-down

Biotin-labeled RNA was synthesized by *in vitro* transcription in the total reaction volume of 20 μl at 37 °C for 3 h with 1 μg pCDNA3-*HMS/HOXC10* 3′UTR using RiboMAX Large Scale RNA Production Systems (P1300) from Promega biotech as per the manufacture instructions along with 2 μM biotin-14-CTP (Invitrogen). Loading solution (95% formamide, 20 mM EDTA, 0.05% bromphenol blue, and 0.05% xylene cyanol) was added to the reaction products and analyzed on an 8% denaturing agarose gel. For RNA pull-down assay, the cell lysate was incubated with biotin-labeled RNA and streptavidin magnetic beads followed by washing, elution, and immunoblotting to detect the RNA-associated proteins. For detecting associated lncRNAs, the RNA was extracted from the eluate using TRIzol and reverse transcribed into cDNA using ThermoScript RT-PCR System kit from Invitrogen as per the manufacture instructions. The qRT-PCR reactions were carried with specific primers to identify associated RNA.

### Subcellular fractionation

To isolate nuclear and cytoplasmic RNAs, U2OS cells were washed and lysed in ice cold 200 μl Buffer A (10 mM Tris pH 8, 140 mM NaCl, 1.5 mM MgCl2, and 0.5% Nonidet P-40) and incubated on ice for 10 min. The cell lysate was centrifuged at 1000*g* at 4 °C for 5 min and the supernatant (representing the cytoplasmic fraction) was mixed with 700 μl of TRIzol reagent for RNA extraction. The cell pellet was resuspended in Buffer B (Buffer A plus 500 mM NaCl, 0.5% NP40) and after centrifugation at 1000*g* at 4 °C for 5 min, the supernatant was discarded, and the pellet (representing the nuclear fracion) was resuspended in 700 μl of TRIzol reagent.

### Data collection

LncRNA expression profile of LUAD was downloaded from TANRIC database (https://ibl.mdanderson.org/tanric/_design/basic/index.html). All of these samples analyzed were from the Cancer Genomic Atlas (TCGA, https://cancergenome.nih.gov/). For LUAD, transcriptional profiles for 420 tumor and 20 normal samples were downloaded (57). We selected those tumor and normal samples for which mRNA and miRNA expression data was available *via* UCSC Genome Browser. The average FPKM values of individual lncRNAs in tumor and normal samples were compared to identify upregulated or downregulated lncRNAs in each cancer. A fold change value of greater than 2 indicated that the expression of the gene is upregulated compared with the normal and the tumor samples, whereas a fold change of less than 0.5 indicated downregulated expression in tumor samples. The correlation analysis between the expression of *HMS* (ENSG00000250742.1) and *HOXC10* gene was done using the online TANRIC tool, which analyzed the expression of tumor samples of the TCGA-LUAD database.

### Statistical analysis

The results are presented as average of n number of independent experiments. Error bars represent standard deviation calculated in Microsoft Excel software as per the formulae: SD = √∑(x_i_ − μ)^2^n, where μ represents the population mean, x_i_ represents each value from the group, n represents the size of the group. For the comparison between two groups, data was analyzed using the Student's *t* test while for comparison between multiple groups, data was analyzed by one-way analysis of variance (ANOVA) followed by post hoc Tukey HSD test using the OneWay_Anova_with_Tukey HSD tool at https://astatsa.com/. The Student *t* test statistic value was calculated in Microsoft Excel software by the formulae: t = (mX − mY)/√(S^2^/n_X_ + S^2^/n_Y_), where X and Y represent the two groups; mX and mY represent the means of groups X and Y, respectively; and n_X_ and n_Y_ represent the sizes of group X and Y, respectively. The critical value of Student’s t distribution at 5% significance level was then determined at the degrees of freedom (df) = n_X_ + n_Y_ − 2. If the absolute value of the *t* test statistics (|t|) was found to be greater than the critical value, then the difference between the groups was considered to be significant (*p* < 0.05). The critical value of the Tukey HSD (Q critical) was established based on number of treatments and degrees of freedom, for significance levels of 0.001, 0.01, and 0.05 (*p*-values) in the studentized range distribution. Next, Tukey HSD Q-statistic value was established for different treatments and compared with the appropriate critical value of the studentized range distribution. If the Tukey HSD Q-statistic was more than Q critical at the desired level of significance (*p*-value), it was concluded that there is significant difference between the compared treatments.

## Data availability

All experimental data is included in the article. Data on lncRNA screening and cloning primers can be requested from the corresponding author, Sandeep Saxena (sandeep@nii.ac.in; sandeepsaxena@mail.jnu.ac.in).

## Supporting information

This article contains supporting information.

## Conflict of interest

The authors declare that they have no conflicts of interest with the contents of this article.
