# Supplementary material for: The lncRNA HMS recruits RNA-binding protein HuR to stabilize the 3′-UTR of HOXC10 mRNA
Source: J Biol Chem. 2021 Jul 22;297(2):100997. doi: 10.1016/j.jbc.2021.100997 (PMC8363838; doi:10.1016/j.jbc.2021.100997)
Supplement: Supplemental Figure S1 [file mmc1.pdf]

Supplementary Figure 1

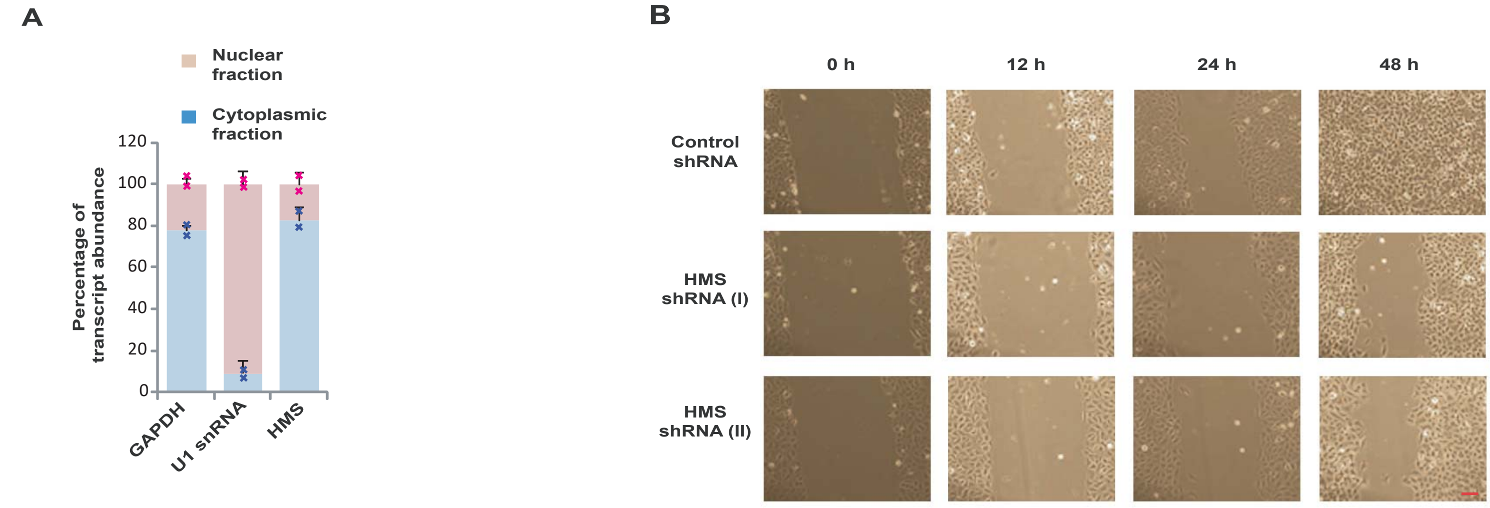

Supplementary Figure 1. *A*, Subcellular fractionation of MG63 cells followed by evaluation of abundance of the indicated transcripts by qRT-PCR in nuclear and cytoplasmic fractions. The bar-graph displays the percentage of the total amount of detected transcripts in different fractions. *GAPDH* and *U1* snRNA serve as controls for cytoplasmic and nuclear fractions, respectively. The data is represented as mean ± SD from two independent experiments. Markers in pink and blue colour point to individual data points of nuclear and cytoplasmic fractions, respectively. *B*, Effect of *HMS* depletion on wound healing assay. Representative fields of data shown in Fig. 2C. *HMS*-depleted U2OS cells were grown to confluence after which a wound was created using a micropipette tip. The extent of wound healing was monitored at the indicated time points. Scale bar, 100 μm. Quantification of wound healing capability observed in shown in Fig. 2*C*.

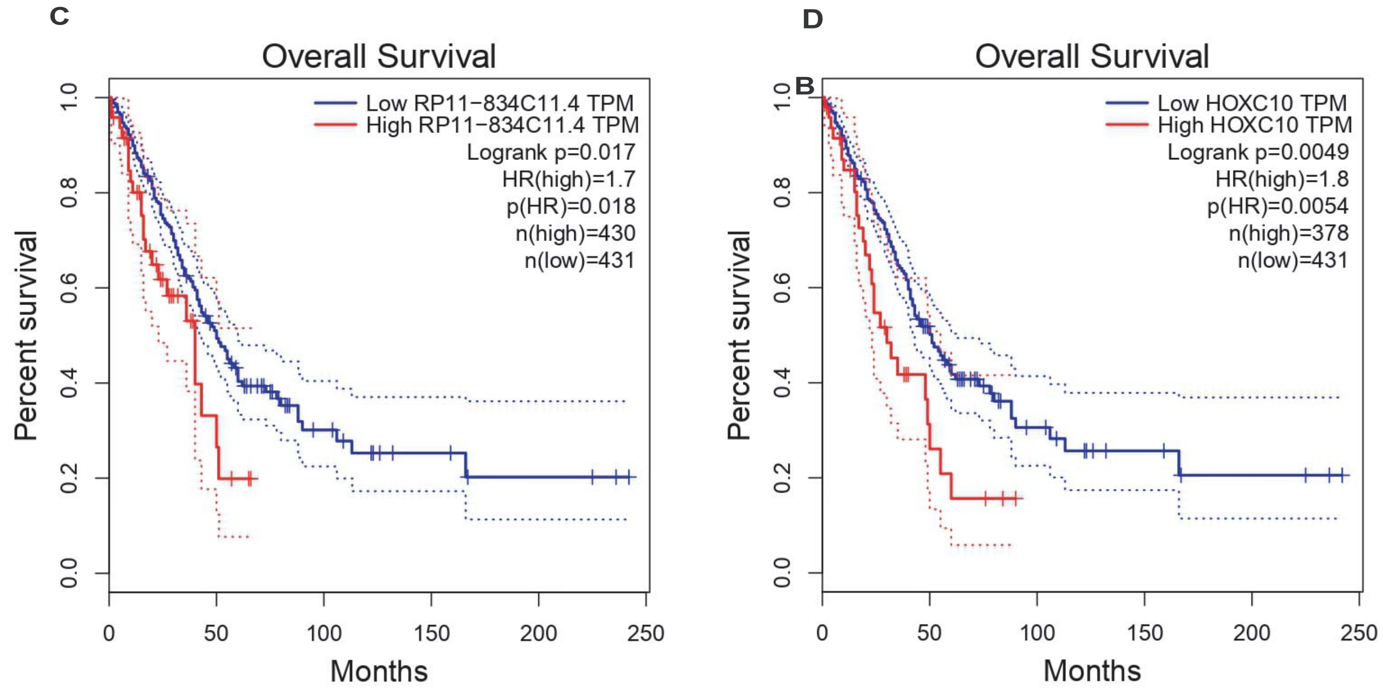

Supplementary Figure 1. *C-D*, Kaplan-Meier estimates of the survival of patients with low or high levels of expression of HMS and HOXC10 in LUAD. The expression and survival information of HMS (C) and HOXC10 (D) was downloaded from GEPIA (Gene Expression Profiling Interactive Analysis) platform. The survival probability with a high group cutoff of 10% and low group cutoff of 90% was calculated at 95% confidence interval.
